# Retro-Aortic Anomalous Coronary Artery (RAC) Sign in a Sexagenarian

**DOI:** 10.7759/cureus.79654

**Published:** 2025-02-25

**Authors:** Hunaina Shahab, Nina Kukar, Nenad Trubelja, Dua Noor Butt, Sajiny John

**Affiliations:** 1 Cardiology, Icahn School of Medicine at Mount Sinai, New York City, USA

**Keywords:** anomalous coronary artery, anomalous left circumflex artery, cardiac computed tomography angiography, rac sign, trans-thoracic echocardiography

## Abstract

Anomalous coronary arteries are congenital anomalies characterized by an abnormal location of the coronary ostium and/or an atypical vascular course. While most cases are asymptomatic and remain undiagnosed, certain variants can be clinically significant, causing symptoms or even sudden cardiac death. A retro-aortic course of the coronary artery is generally considered to be benign, though rare instances have been reported to cause myocardial ischemia. This course can be detected on transthoracic echocardiogram (TTE) as a retro-aortic anomalous coronary artery (RAC) sign. The RAC sign is strongly associated with the detection of a retro-aortic coronary anomaly on cardiac computed tomography (CT) scans. Given its high specificity, its identification on TTE can be reliably documented as highly suggestive of an anomalous coronary artery in echocardiography reports. We report the case of a 67-year-old woman presenting with nocturnal syncope. TTE reported a tubular echogenic density in the atrioventricular (AV) groove. Coronary CT angiography (CCTA) showed an anomalous left circumflex coronary artery (LCx) arising from the right coronary cusp taking a retro-aortic course to the left AV groove. There were no high-risk anatomical features of the anomalous LCx or any significant coronary artery stenosis. Holter monitor revealed sinus pauses of up to nine seconds, correlating with her symptoms. She was diagnosed with sick sinus syndrome and a permanent pacemaker was implanted. She remained asymptomatic thereafter. The TTE finding, labeled as the RAC sign, correlated well with the anomalous LCx on the CCTA. The anomalous LCx was noted to be incidental and likely benign.

## Introduction

Congenital coronary artery anomalies (CCAAs) refer to a group of congenital conditions in which any of the three epicardial coronary arteries have an abnormal origin or course [1]. Although the true prevalence of CCAA remains unknown largely due to the absence of clinical indication for testing, studies have shown a low prevalence of <1% in the general population [2]. Although evidence indicates that anomalous right coronary artery (RCA) is about six times more common than anomalous left coronary artery, anomalous left coronary artery appears to account for up to 85% of sudden cardiac deaths associated with CCAA [3,4]. After hypertrophic cardiomyopathy, CCAA is the second highest cause of sudden cardiac death amongst US athletes accounting for 17% of the cases, however, only 2.3% were attributed to anomalous RCA [5].

CCAA can be found on transthoracic echocardiogram (TTE), especially in the pediatric population [1]; however, suboptimal echocardiographic windows, technical challenges, patient cooperation, and inadequate operator experience may limit diagnosis [6,7]. With the advancing child's age, it becomes increasingly challenging to evaluate coronary arteries on TTE [6]. Coronary computed tomography (CT) angiography (CCTA) is the primary imaging modality for the diagnosis of CCAA [1,7]. CCTA is particularly valuable for diagnosing CCAA when coronary origins are not clearly visualized, or an anomalous origin is suspected on TTE [6]. Furthermore, even when TTE provides a definitive diagnosis of CCAA, CCTA offers superior capability in assessing additional anatomical details [6]. CCTA can provide detailed anatomical characterization with an assessment of the relationship of coronary arteries with surrounding cardiac and noncardiac structures, precise three-dimensional visualizations, and identification of high-risk features of CCAA [1].

The retro-aortic anomalous coronary artery (RAC) sign, first named in 2018 as a TTE finding, was described as a highly echogenic tubular structure on an echocardiogram, located on the atrial side of the atrioventricular (AV) groove [8]. This finding correlated with an anomalous coronary artery taking a retro-aortic course in the AV groove with a sensitivity of 63% and a specificity of 94% [8], with the lower sensitivity explained by possible dismissal of this finding as an artifact by cardiac imagers when found on TTE [8]. The RAC sign is strongly associated with the detection of retro-aortic coronary anomalies on cardiac CT scans. Given its high specificity, its identification on TTE can be reliably documented as highly suggestive of an anomalous coronary artery in echocardiography reports [8].

Here, we present the case of a 67-year-old woman with a history of nocturnal syncopal episodes. TTE showed a tubular echogenic density in the left AV groove near the mitral valve. Subsequently, CCTA showed an anomalous left circumflex coronary artery (LCx) arising from the right coronary cusp (RCC) taking a retro-aortic course to the left AV groove. The TTE finding was deemed to be the RAC sign.

## Case presentation

**Figure 1:**
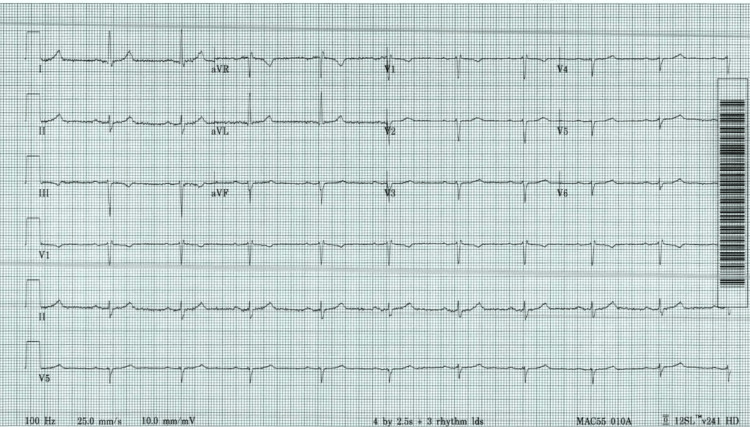
ECG showing normal sinus rhythm at the rate of 60 beats per minute, leftward axis, prolonged PR (204 milliseconds), poor R-wave progression, and non-specific ST/T changes.

A 67-year-old woman, with controlled type II diabetes mellitus and hypertension for the past 10 years, presented to the cardiology clinic with one-month history of nocturnal syncopal episodes. She denied chest pain, dyspnea, or palpitations. On examination, she was vitally stable with a heart rate of 66 beats per minute and a blood pressure of 138/82mmHg. On examination, she had normal jugular venous pressure, normal S1 and S2 without audible murmurs, absence of lung crepitations, and absence of pedal edema. She was taking losartan-hydrochlorothiazide (100mg-12.5mg once daily) for hypertension and metformin (500mg twice a day) for diabetes mellitus. Her electrocardiogram (ECG) showed a normal sinus rhythm at the rate of 60 beats per minute, prolonged PR interval (204 milliseconds), leftward axis deviation, poor R-wave progression, and nonspecific ST/T changes as shown in Figure 1. These ECG findings hinted at a possible underlying conduction abnormality which could potentially explain her symptoms, therefore further cardiac workup including a Holter monitor was done.

Her blood workup, including hemoglobin, creatinine, blood glucose, electrolytes, liver function, and thyroid panel, was unremarkable. Her blood pressure was well controlled, and no hypotension was observed with her antihypertensive medication dose to explain her symptoms. Her blood glucose was controlled, and no hypoglycemia was noted to explain her symptoms. 

TTE revealed a normal left ventricular size and ejection fraction (65%) and a normal right ventricular size and function. A tubular echogenic density was noted to be parallel to the mitral valve as seen in the apical four-chamber view in Videos 1, 2, apical two-chamber view in Video 3, and apical three-chamber view in Video 4.

**Video 1:** Transthoracic echocardiogram showing the apical four-chamber view. Apical four-chamber view showing normal left ventricular size and function, normal right ventricular size and function and a tubular echogenic density in the left atrioventricular groove in the vicinity of the mitral valve. The mitral valve appears to be structurally normal.

**Video 2:** Transthoracic echocardiogram showing the apical four-chamber view with color Doppler. Apical four-chamber view with color Doppler applied across the mitral valve showing normal left ventricular size and function, normal right ventricular size and function and a tubular echogenic density in the left atrioventricular groove in the vicinity of the mitral valve. There was no significant mitral valve regurgitation and no significant turbulence of color Doppler, indicating the absence of mitral stenosis.

**Video 3:** Transthoracic echocardiogram showing the apical two-chamber view. Apical two-chamber view showing normal left ventricular size and function. A tubular echogenic density was noted to be in the left atrioventricular groove in the vicinity of the mitral valve. The mitral valve appeared structurally normal with normal opening without significant stenosis.

**Video 4:** Transthoracic echocardiogram showing the apical three-chamber view. Apical three-chamber view showing normal left ventricular size and function. A tubular echogenic density was noted in the left atrioventricular groove in the vicinity of the mitral valve. The mitral valve appeared structurally normal with normal opening without significant stenosis.

**Figure 2:**
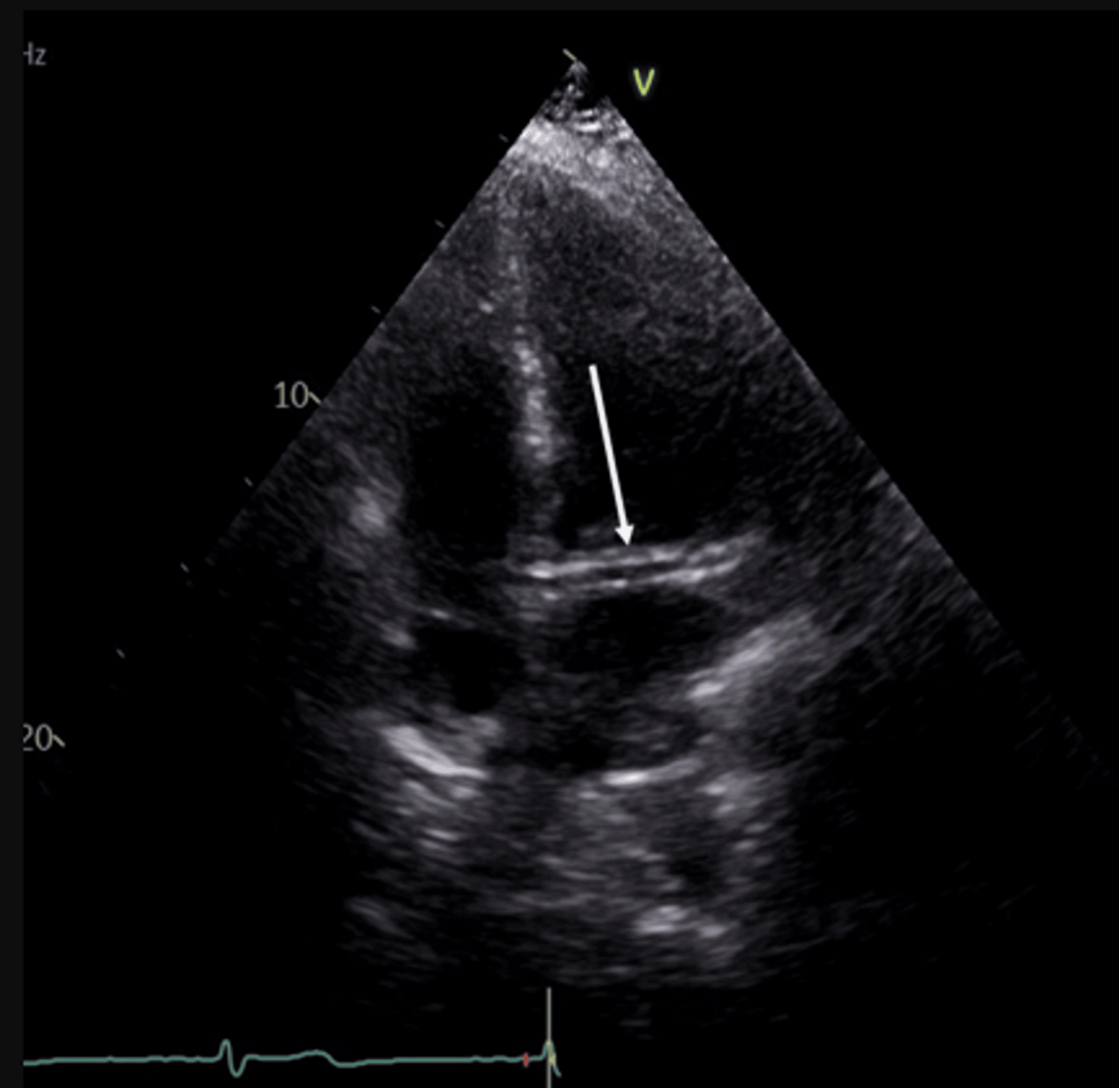
Transthoracic echocardiogram four-chamber view showing the tubular echogenic density parallel to the mitral valve (arrow).

The echo density was noted to be below the aortic valve level in the short-axis view in Video 5. Figure 2 shows the tubular echogenic density parallel to the mitral valve.

**Video 5:** Transthoracic echocardiogram showing modified short-axis view at the level of the aortic valve. A tubular echogenic density appeared below the aortic valve level in this modified short-axis view.

**Figure 3:**
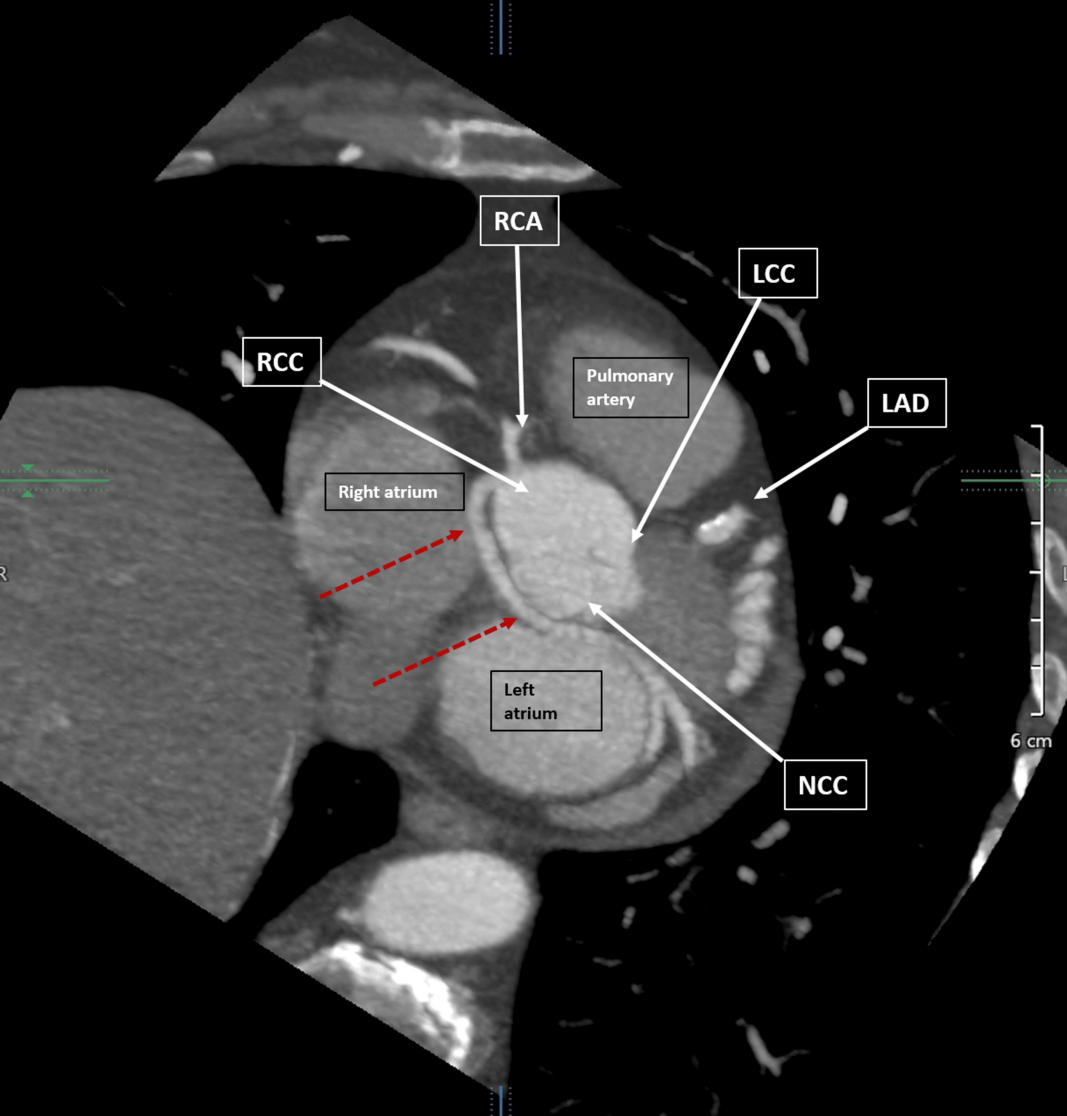
Coronary CT angiography (modified axial view) showing an anomalous left circumflex coronary artery (red dashed arrows) originating from the right coronary cusp and taking a retro-aortic course to the left atrioventricular groove. Coronary CT angiography (modified axial view) showing anomalous left circumflex coronary artery (LCx) originating from the right coronary cusp (RCC) and taking a retro-aortic course to the left atrioventricular groove (red dashed arrows). The ostium of the LCx is separate from the ostium of the right coronary artery (RCA). The left anterior descending coronary artery (LAD) is seen in the anterior interventricular groove with <25% stenosis due to calcified plaque. Also noted in the figure are the left coronary cusp (LCC) and the noncoronary cusp (NCC).

**Figure 4:**
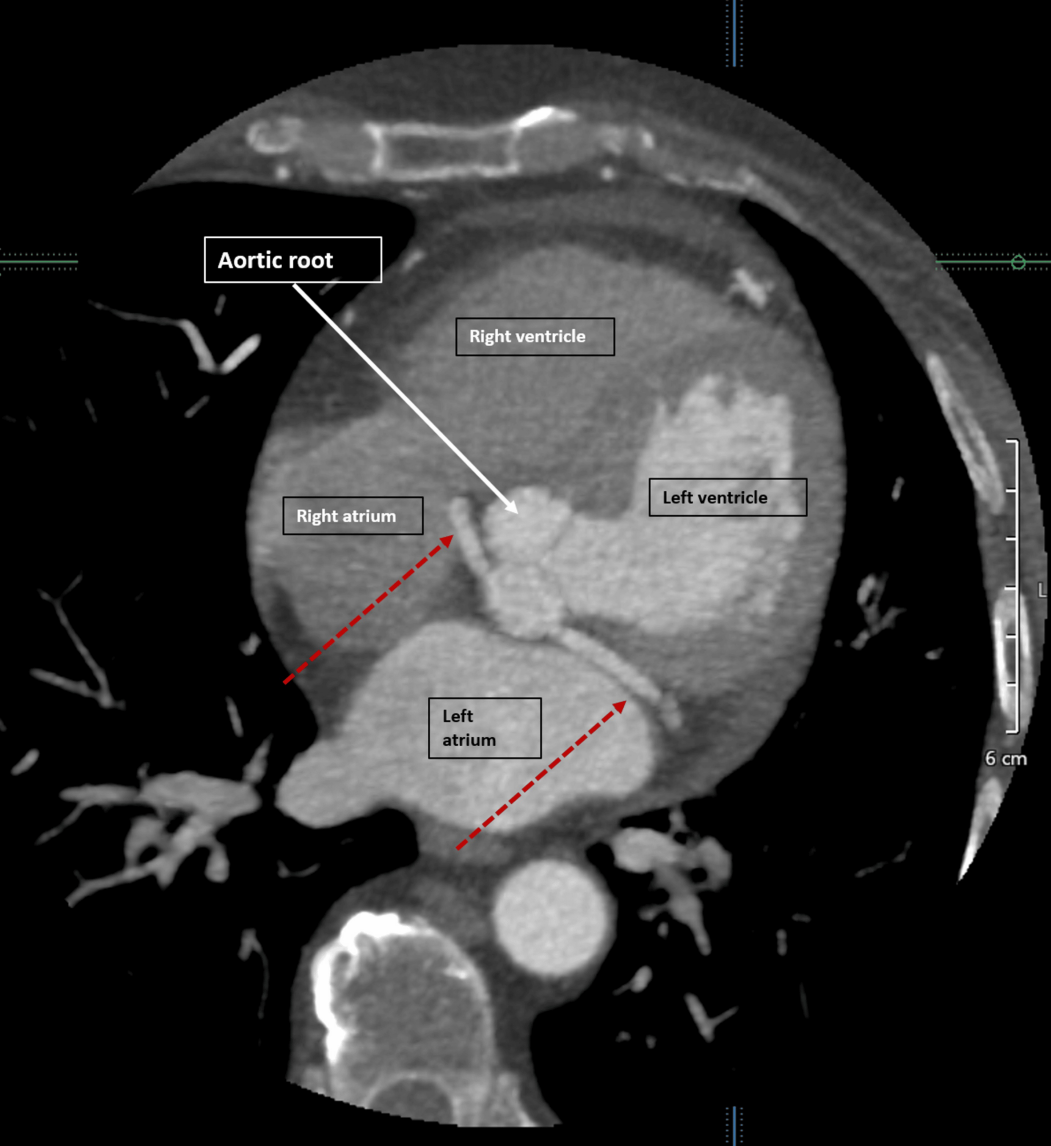
Coronary CT angiography (axial view) showing anomalous left circumflex coronary artery with a retro-aortic course to the left atrioventricular groove (red dashed arrows).

A CCTA was done which showed a dominant LCx originating anomalously from the RCC, following a retro-aortic course towards the left AV groove as shown in Figures 3, 4.

**Figure 5:**
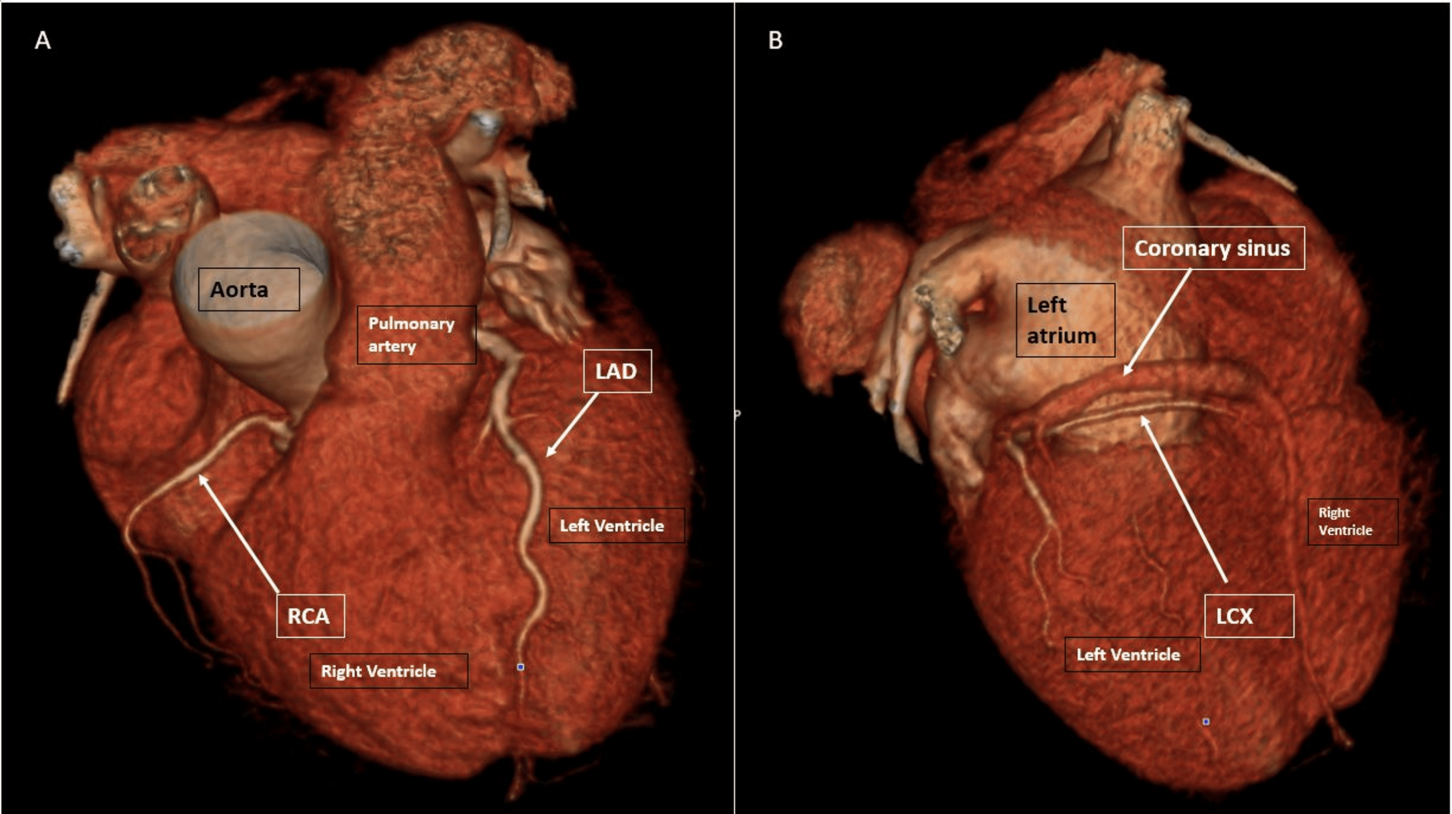
Three-dimensional volume-rendered cardiac CT images. (A) Normal course of the left anterior descending artery (LAD) to the anterior interventricular groove and the right coronary artery (RCA) to the right atrioventricular groove. (B) Dominant left circumflex artery (LCx).

The RCA originated normally from the RCC with an ostium separate from the anomalous LCx. The LAD originated normally from the LCC. The calcium score was 1 with <25% stenosis in the mid-LAD due to calcified plaque. There was no stenosis or significant atherosclerotic plaque in any other coronary artery. Three-dimensional volume-rendered cardiac CT images (Figures 5A, 5B) show the course of the coronary arteries. The tubular echo density seen on the TTE coincided with the anomalous LCx coursing with a retro-aortic course through the AV groove and was labeled as the RAC sign.

**Figure 6:**
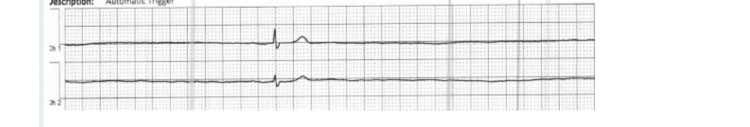
Holter monitor showing sinus pauses.

Holter monitor uncovered sinus bradycardia with intermittent first- and second-degree AV block, with up to 9.3-second pauses (Figure 6) correlating with her symptoms. She reported experiencing syncope at the same time as the pause was detected on the Holter monitor. These pauses lead to periods of cerebral hypoperfusion, which explains her syncopal symptoms. 

Based on the clear documentation of syncopal episodes in the presence of pauses on the Holter monitor, absence of high-risk features of the anomalous LCx or obstructive coronary artery disease, and normal blood work parameters, she subsequently underwent a dual chamber permanent pacemaker (PPM) implantation and remained asymptomatic thereafter. Her PPM is under regular follow-up and monitoring by an electrophysiologist. She is also under close follow-up by a cardiologist to monitor any further symptoms.

## Discussion

With the growing use of advanced cardiac imaging techniques, the detection rate of congenital coronary artery anomalies (CCAAs) in adults is increasing [1]. The most common type of CCAA is the LCx arising from the RCA or the RCC, with less common ones including the left coronary artery, or the RCA coming off a different sinus of Valsalva or another coronary artery, or from an ectopic site from the aorta [9]. Most patients with CCAA are asymptomatic, however, some variants are clinically significant causing either symptoms or sudden cardiac death [10]. The high-risk anatomic features of CCAA include inter-arterial course (between the aorta and pulmonary artery), intramural course (within the wall of the aorta), slit-like ostium, acute angle of origin and proximal narrowing of the anomalous artery [10,11]. These high-risk features can predispose to a higher risk of sudden cardiac death especially on exertion [10,11]. However, some studies suggest that this risk may be overestimated due to a reporting bias as many cases are derived from those who presented with fatal events [10,12,13]. 

In our case, there were no high-risk anatomic features of the anomalous left circumflex artery that could predispose to a higher risk of sudden cardiac death. Furthermore, no evidence of obstructive coronary artery disease could contribute to the patient’s symptoms. The absence of symptoms up until the age of 67 years, the Holter monitor results, and the improvement in symptoms after the PPM implantation further reinforced the benign nature of this incidental finding of the anomalous LCx.

The clinical implication of the RAC sign is not certain [8]. Although the retro-aortic anomalous coronary artery is mostly understood to be benign [8,14], some studies have reported it as a cause of myocardial ischemia needing surgical correction [15]. Some other reports have described the compression of the anomalous LCx leading to myocardial infarction following aortic and mitral valve replacement, necessitating surgical revascularization [16]. Differential diagnosis of the RAC sign includes artifacts, valvular or annular calcifications [17], and catheters or leads [8]. Therefore, it is imperative that whenever this sign is present on TTE, it is well documented to prevent potential future complications or clinical mismanagement due to misdiagnosis.

Guidelines give class 1 recommendations for surgery in the case of the left coronary artery arising from the right coronary in the presence of symptoms or diagnostic evidence of ischemia [18]. A recent study demonstrated that the anomalous LCx was considered a benign finding, with little to no consequences for physically active people, or recreational athletes [19]. The most recent guidance statement addressing competitive sports participation for athletes with cardiovascular abnormalities advises that competitive athletes with benign coronary artery anomaly subtypes that are not associated with myocardial ischemia, including the anomalous circumﬂex from the right aortic sinus or the RCA with retro-aortic course, can participate in competitive sports [20].

## Conclusions

The retro-aortic course of the anomalous LCx was clearly identified on TTE as the RAC sign in our case. This was an incidental finding and was unlikely to have contributed to the patient's symptoms, as evidenced by symptom resolution following PPM insertion. Our case adds to the growing body of literature supporting the notion that a retro-aortic anomalous coronary course is generally a benign entity. Given the high specificity of the RAC sign, its presence on TTE should be reliably documented as a strong indicator of an anomalous coronary artery, warranting further imaging with CCTA or coronary angiography as clinically appropriate. Subsequent management should be guided by the patient’s symptoms and overall clinical presentation. Further research is needed to better understand the long-term clinical outcomes of patients with a retro-aortic anomalous coronary course that is deemed benign at the time of diagnosis.
